# From chloroquine to artemisinin-based combination therapy: the Sudanese experience

**DOI:** 10.1186/1475-2875-5-65

**Published:** 2006-07-31

**Authors:** EM Malik, TA Mohamed, KA Elmardi, RM Mowien, AH Elhassan, SB Elamin, AA Mannan, ES Ahmed

**Affiliations:** 1 National Malaria Control Programme, P.O. Box: 1204, Tel +249 183 776809, Khartoum, Sudan; 2 College of Medicine, University of Juba, Sudan

## Abstract

**Background:**

In Sudan, chloroquine (CQ) remains the most frequently used drug for falciparum malaria for more than 40 years. The change to artemisinin-based combination therapy (ACT) was initiated in 2004 using the co-blister of artesunate + sulfadoxine/pyrimethamine (AS+SP) and artemether + lumefantrine (ART+LUM), as first- and second-line, respectively. This article describes the evidence-base, the process for policy change and it reflects the experience of one year implementation. Relevant published and unpublished documents were reviewed. Data and information obtained were compiled into a structured format.

**Case description:**

Sudan has used evidence to update its malaria treatment to ACTs. The country moved without interim period and proceeded with country-wide implementation instead of a phased introduction of the new policy. The involvement of care providers and key stakeholders in a form of a technical advisory committee is considered the key issue in the process. Development and distribution of guidelines, training of care providers, communication to the public and provision of drugs were given great consideration. To ensure presence of high quality drugs, a system for post-marketing drugs surveillance was established. Currently, ACTs are chargeable and chiefly available in urban areas. With the input from the Global Fund to fight AIDs, Tuberculosis and Malaria, AS+SP is now available free of charge in 10 states.

**Conclusion:**

Implementation of the new policy is affected by the limited availability of the drugs, their high cost and limited pre-qualified manufacturers. Substantial funding needs to be mobilized by all partners to increase patients' access for this life-saving intervention.

## Background

Malaria is a leading cause of morbidity and mortality in Sudan resulting in an estimated 7.5 million cases and 35,000 deaths annually [1]. Possible contributing factors leading to this grave situation include floods, draught, famine, widely extended irrigated schemes without enough consideration to health component and population movement (internal displacement and influx of refugees). The situation may be further aggravated by insecticide resistance [2] and the spread of *Plasmodium falciparum *resistant strains [3].

Chloroquine (CQ) has been the most frequently used drug in Sudan as a first line for years. The drug was available through public, private and not-for-profit pharmacies. A recent study showed that 85.6% of severe malaria patients admitted to five hospitals in central Sudan received CQ before hospitalization [4] and that CQ was also the commonly prescribed drug by health care providers [5].

In Sudan, malaria control was based for decades on vector control, through spraying with insecticides. In the late 1990s, great efforts were directed to improve malaria case management and this was included in the development of the Roll Back Malaria (RBM) strategic plan in 2001. Case management requires the provision of prompt, effective and safe treatment to malaria cases [6]. Using the results of research on resistance to CQ, Sudanese policy-makers updated their national malaria treatment guidelines in 2004 to artemisinin-based combination therapy (ACTs), both as first- and second-line treatment for the management of uncomplicated falciparum malaria. This case study aims to provide the evidence-basis and to describe the process for change and the experience of one year implementation.

Information and data tackled here were collected using two methods. Published antimalarial drugs efficacy studies carried out in Sudan between 1975 and 2005 were identified through searches of on-line databases (PubMed Medline – using the address: ). Published data not on-line were also identified and copies from authors were collected when possible. Official unpublished documents related to the drug policy issue, procurement or manufacturing of the anti-malarial drugs were obtained from the National Malaria Control Programme (NMCP), General Directorate of Pharmacy (GDP) and the Central Medical Supply (CMS). Data of regional and national conferences or meetings were also obtained in soft or hard copies either directly from the organizers or from websites.

## Case description

### Efficacy studies supporting change from CQ to ACT

From 1975 to 2005, many drug efficacy studies were carried out in Sudan, several published in international and national scientific journals. Table 1 presents the proportion of in vivo parasitological treatment failure for various antimalarials used as monotherapy or combination therapy for the treatment of uncomplicated falciparum malaria in Sudan. Since the 1970s, CQ resistance has been documented and has since increased and spread all over Sudan [7-15]. Limited studies of sulfadoxine-pyrimethamine (SP) efficacy were only carried out in 2000, although it had been used as a second-line treatment for a long time [13-18]. Efficacy of combination therapy started by testing the efficacy of (CQ+SP) combination in four sites. High level of resistance (15.5 – 36.0%) was determined in three of them [17]. Results of ACTs studies started in 2003 and became available (late in 2003 or early in 2004) for the National Malaria Control Programme (NMCP) before publication. The combination of artesunate plus SP (AS+SP) was tested in 10 areas with no reported failure in seven of them, <1.0% failure rate in two and up to 8.8% failure rate in one site [18,21-24]. Artesunate plus amodiaquine (AS+AQ) was tested in southern and western Sudan with a failure rate of 1.0 to 7.3% respectively [20,22]. Artemether plus lumefantrine (ART+LUM) was tested in two sites with no reported failure rate [24].

**Table 1:** Susceptibility of *P. falciparum *to antimalarials in Sudan, 1975–2005.

**Period**	**Drugs tested**	**Regions**	**Protocol used**	**follow-up (days)**	**Failure rate (%)**	**References number**
1975–97	CQ	central	Standard WHO in-vivo test	7–28	0.8–25.0	7, 10
		eastern			43.0–48.0	8, 9
1998–03	CQ	central	WHO 1996	14–28	25.0–75.0	11, 12
		eastern	WHO 1996	28	76.9	13
		southern	WHO 1996, EANMAT 1999	14–28	11.5–93.9	14, 15
		north Sudan	WHO 1996	14	32.0–70.0	UPD
	SP	eastern	WHO 1996	28	0.0–19.4	16, 17
		southern	WHO 1996, EANMAT 1999	14–28	0.0–69.9	15, 14
		north Sudan	WHO 1996	28	1.3–7.8	UPD
	AQ	southern	WHO 1996, EANMAT 1999	14	5.9–25.2	14, 15
	MQ	eastern	WHO 1996	28	7.5	20
	Q	eastern	WHO 1996	28	9.4–10.7	13
	CQ+SP	eastern	WHO 2003	28	2.5–36.0	17, UPD
		central	WHO 1996	28	15.5–20.0	UPD
	AS+SP	western	WHO 2003	28	8.8	21
		southern	WHO 2003, 1996	28–42	0.0–0.9	22
		central	WHO 2003	28	0.0	UPD
	AS+AQ	western	WHO 2003	28	7.3	21
		southern	WHO 2003, 1996	42	1.0	22
2004–05	AS+SP	eastern	WHO 2003	28	0.0–0.7	18, 23
		southern	WHO 2003, 1996	28–42	0.0	23
		central	WHO 2003	28	0.0	23, 24, UPD
	ART+ LUM	southern	WHO 2003	28	0.0	UPD
		central	WHO 2003	28	0.0	24

UPD: unpublished data

### Process for change

**Figure 1:**
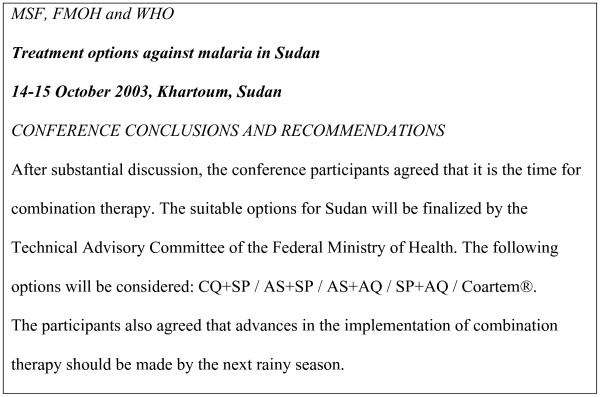
Treatment options against malaria in Sudan" conference conclusions and recommendations.

In 1998, the NMCP organized an important workshop on malaria treatment to develop national treatment guidelines, with CQ as first-line. In 2001, the NMCP established a technical advisory committee (TAC) to assist the Federal Ministry of Health (FMOH) in decisions related to first-line treatment of malaria, in view of available data about CQ resistance. The TAC is constituted of 40 members, representing clinicians, pharmacists, policy makers, UN agencies and academic and research institutes with support, from time to time, from WHO consultants. The TAC refused the suggestion from the NMCP to change the policy from CQ to CQ+SP because of the lack of evidence. Secondly, the decision to change from CQ to ACTs was taken based on national and international evidence. An important landmark in the process of change has been the "treatment options against malaria in Sudan" conference initiated by Médecins Sans Frontières (MSF) and arranged jointly between FMOH, WHO and MSF in October 2003. The conference reviewed the most recent evidence and the experience of other countries and it generated recommendations, which are reflected in Figure 1. Immediately after the conference, a small committee (members of TAC who participated in the conference) was nominated by the NMCP to select the drugs to be used as first- and second-lines in Sudan. The work of this committee then reviewed and approved by the TAC retaining AS+SP as first-line and ART+LUM as second-line treatment.

The policy was then endorsed by Undersecretary of Health, who communicated the new malaria treatment policy to all concerned bodies of FMOH (General Directorate of Pharmacy, Central Medical Supply, Integrated Management of Childhood Illnesses programme, etc) and to major partners agencies (WHO, UNICEF, MSF, etc). Subsequently, the national guidelines for treatment of malaria developed by NMCP staff, endorsed by the TAC, revised by local experts and WHO consultants and edited. Further, the guideline was piloted in 25 sites (in five states) to obtain feedback from providers. Remarks were taken into consideration when the final version was developed.

### Implementation of change

**Figure 2:**
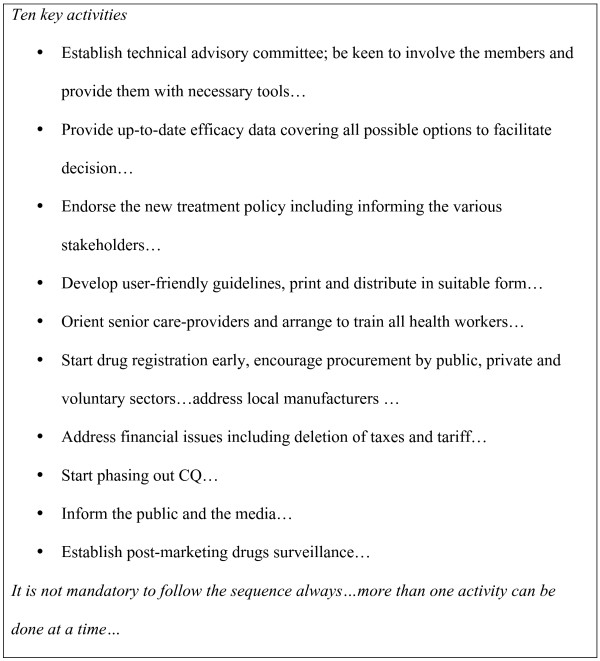
Key activities in the processing of changing from CQ to ACTs.

The NMCP worked simultaneously on many issues related to the implementation of the new treatment policy (Figure 2). The new guidelines were summarized as booklets and wall charts, both in Arabic and in English, and enough copies were printed and distributed at different levels with support from FMOH, WHO, NGOs and private companies. Orientation sessions aiming to launch the new treatment guidelines were carried out at state capitals for physicians, pharmacists, Ministry of Health Heads of Departments and policymakers in 20 states. Each session (2–3 hours) consisted of a formal presentation, the distribution of guidelines, an open discussion and showing samples of the recommended drugs. This was followed by a 3-day training session for medical doctors, medical assistants, pharmacists, assistant pharmacists and community health workers. The training, which is still going on, was started by the training of trainers assigned by different states using a training manual. These trainers arranged for training in their respective states with the assistants from NMCP staff and senior physicians. The GDP announced through its website for companies to submit for registration according to pharmaceutical regulations and specifications stated in the treatment guidelines (eg a co-blister of AS and SP). The NMCP also addressed local manufacturers and encouraged them to change from CQ to the new drugs. A co-blister of AS+SP from two of them is becoming available for assessment by GDP in October 2005. One of the assessed products has been registered and distributed and the registration of the other is bending on fulfilment of certain technical and administrative requirements. A system for drug post-marketing surveillance was arranged in collaboration with the national drug laboratory for quality assurance. Official inspector (pharmacist from National Malaria Control Programme – Sudan) coordinating collection from several states and was responsible for sample coding and transport to the National Drug Quality Control Laboratory at Khartoum for analysis. Samples were analyzed according to the international pharmacopoeia and Chinese pharmacopoeia to assess the quality of the products. International pharmacopoeia is the first choice for work in the laboratory but Chinese pharmacopoeia was used for dissolution test as that test has not been developed yet in the international pharmacopoeia.

The experience of other countries was taken into consideration and the Zanzibar experience with the use of AS+AQ was shared through a visit (arranged by the Malaria Consortium) of the Zanzibar RBM coordinator to Sudan. Communicating the change to the public was implemented progressively. Broadcasting messages about malaria treatment and ACTs through national radio was implemented since September 2005. The communication programme consists of four messages per day to be delivered for two weeks, followed by a break for one week and then the message is delivered again for two weeks, and so on. This was supported by WHO and the National Broadcasting Corporation. The forecasting is targeting the best time for maximal audience.

### What about chloroquine?

**Figure 3:**
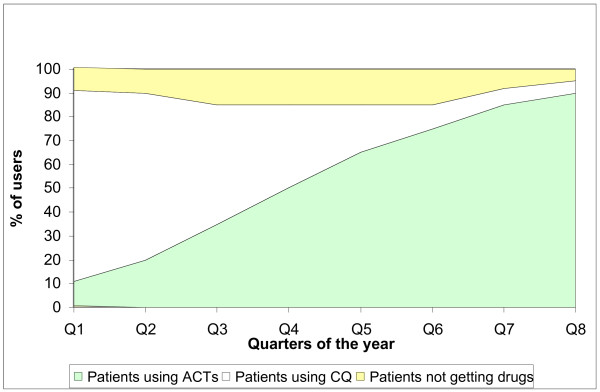
Phasing out of CQ as response to increasing use of ACTs over 2 years (hypothetical figures).

The plan is for CQ to be phased out within two years (Figure 3). CQ is widely available, cheap, providers and care-givers still rely on it. On the other hand, the availability of the new recommended drugs is still limited and they are not affordable. In January 2005, to promote phasing out, the GDP circulated a letter to manufacturers and importers of CQ to reduce their quantities by 40%. By January 2006, the manufacturing and importation of new CQ in form of tablets and vials was stopped completely. This was initiated by a letter from NMCP to GDP which in turn informed both the manufacturers and importers of CQ in addition to related authorities. Still one can get CQ from the circulating drugs in the market.

### Experience of one year implementation

As regards to first-line treatment (AS+SP), four companies have so far registered their products in Sudan. All of them produce AS+SP as co-blister and started distribution in the private sector. In addition, a single manufacturer has registered ART+LUM and another artesunate suppositories. Two drug factories started the process of local manufacturing of AS+SP and the product of one of them is becoming available at the drugs distribution outlets. The new recommended medicines have been available through public, NGOs and private sectors since August 2004. The total amount of AS+SP strips and of ART+LUM tablets procured and distributed by all sectors, from August 2004 to October 2005, was 4,095,000 and 495,000, respectively. The fund used for procurement of AS+SP came from Central Medical Supply (69.0%), private companies (23.4%) and UN agencies and NGOs (7.6%). The coverage with ACTs is expected to increase with the additional input from the Global Fund to fight AIDS, Tuberculosis and Malaria (GFATM). Treatment of malaria in Sudan at public sector level is not free and a system of full cost recovery has been in operation since the early 1990s, as part of the revolving drug fund (RDF). The retailed price of the drug, include the known international cost in addition to 62% (this include 27% as taxes and 35% as private benefit) when the drug has been procured and distributed by the private sector. Thus >90% of the money needed for the procurement is provided by malaria patients. The price for AS+SP ranged between $2.4 and $4.4 per adult treatment and between $2.0 and $3.2 per child. One tablets of ART+LUM cost the patient $0.4 and the cost varies between $2.4 and $9.6 per treatment, respectively for the youngest child and for an adult. Both drugs are available at urban setting from which medical assistants and senior nurses working at dispensaries in rural areas can get the drugs. A system for post-marketing drug surveillance was established in collaboration with the GDP. The case management staff of the NMCP collects the drugs from different part of the Sudan and the drug quality laboratory at GDP conducts the necessary tests. Two rounds targeting AS+SP have been completed so far.

## Discussion

### Evidence for change

Confirmed cases of CQ resistance in Sudan were first reported in 1978 [7]. The first evidence to support the change from CQ to alternative drugs was compiled and presented at the workshop on malaria treatment in Khartoum in 1998. As a result, malaria treatment guidelines were developed to promote the rational use of antimalarials, including CQ and SP, with emphasis on regular monitoring of the efficacy of CQ. From 1998 to 2003, treatment guidelines were developed and distributed all over the country, training targeting different providers was carried out and sentinel sites to monitor CQ and SP efficacy were established with support from WHO. Later in 2001, a TAC was formed with regular meetings. As in many countries in Africa [25-29], Sudan has used evidence to update its malaria treatment from CQ to ACTs. This was recognized by the Council on Health Research for Development – COHRED [30]. By the end of 2003, overwhelming evidence showed that CQ was no longer suitable for uncomplicated malaria treatment in Sudan [3,12,14,15,31], Furthermore, SP treatment failure ranged between 1.9% and 7.8%. Recent studies showed, CQ+SP treatment failure rate ranging between 2.5 to 36.0% [17] in contrast to high response rate to ACTs – (AS+SP and AS+AQ) [21,22]. In addition, researchers from NMCP and other institutes provided supportive data after the launching of the new treatment guidelines [18,21,23,24]. Sudan moved from CQ to ACTs without an interim period, in contrast to other countries in Africa [28,29,32,33]. Sudan also proceeded with country-wide implementation instead of a phased introduction of the new policy. Despite the evidence of CQ resistance in many WHO Eastern Mediterranean Region [3], Sudan was the first country in the region to shift to ACTs.

### Factors affecting the decision

Changing drug policy is complex. Limiting factors are high cost, limited knowledge and lack of public awareness on the effectiveness and safety of ACTs in addition to operational issues [34-36]. Changing from CQ in particular is challenging as stated by other authors [29]. CQ is widely available, easy to use and cheap. These factors delayed the decision in Sudan despite the fact that all the TAC members were convinced of the necessity to change from CQ to an alternative drug very early in 2002. Nevertheless, Sudan has changed its malaria treatment protocol in 2004 to provide artemisinin-based combination therapy as first-line (AS+SP) and second-line (ART+LUM) treatment. The conference arranged by MSF-France (treatment options against malaria in Sudan) and the technical support from WHO played major role in solving this concern. TAC members recognized the high benefits of providing effective drugs to those who are suffering, despite the high costs of the drugs. ACTs may cost up to 20 times more than the currently available antimalarials [36], but considering the factors that determine the effectiveness of malaria treatment policy [35], the direct cost of implementing ACTs (AS+AQ) for confirmed malaria cases could be up to 53% less expensive than using CQ and Q for clinically diagnosed cases as documented in Senegal [37]. Several studies have documented that continued use of CQ is contributing to increase hospital attendance and admission, malaria-associated mortality [38,39], severe malarial anaemia [39], and malaria case fatality rate may decreased with the provision of effective treatment [40]. A recent study in KwaZulu Natal, South Africa, also documented that implementation of ACTs reduced total expenditure on malaria services [41]. Some of these factors led to the decision of phasing out of CQ rather than abrupt cessation of monotherapy in order to avoid a supply shortage while an increasing proportion of people is being convinced that CQ is no longer the suitable treatment option and new medicine become available.

### Challenges to implementation of change

Faced with the dilemma of comparing the newly recommended drugs with CQ, the population prefers drugs which are cheap, easy to use, readily available and safe for all age groups [42]. Successful strategies to change behaviour need to include the use of rigorous evidence, involving scientist in the decision-making process, focusing on communication, receiving support from credible partners (WHO), utilizing regional approaches rather than focusing solely on the home country, and a better understanding of the political system [43]. In Sudan, it was decided to have the first-line as co-blister rather than each drug separately. Negative aspects of monotherapy, which may affect prescriber's and consumer's practice, include: the provider not prescribing both drugs, the patient not taking both drugs because of the price or not accepting to use both drugs. Policy makers at GDP were highly concerned about the availability of ACTs and their stability in hot wheather, such as exists in Sudan. As no company at time of launching the new policy was qualified by WHO to provide co-blister of AS+SP, the registration of the recommended first-line drug was difficult and further complicated by results of post-marketing surveillance of some company products which showed sub-standard stability. A recent household survey carried out in 10 states to provide base-line data for GFATM support showed limited availability of ACTs and hence treatment of malaria still relied on CQ (65.6%). The treatment with ACTs was reported to be only 10.5% on average. Higher rates were seen in urban areas (18.1%) and the lowest in rural areas (5.6%). This is similar to the Zambian experience, even if ART+LUM was made available free-of-charge in Zambia [44].

In Sudan, drugs are available to patients through the RDF in the public sector as well as in the private and NGOs sectors. Drugs provided through the public sector were having relatively low price compared with the private sector (almost 30–50% lower). The difference in price is due to bulk purchase and exemptions from high taxes and tariff (almost 27%). High cost was found to delay treatment-seeking in Sudan [4,45]. Therefore, the public sector has to take the lead in ensuring access and the NMCP should develop the strategies recommended to improve the affordability and financing of ACTs [46]. The release of round-two of GFATM support, in addition to procurement through NGOs and UN agencies, give some hope in improving availability and affordability, considering that the estimated cost of ACTs is beyond the country resources [47]. By January 2006, a total of 995,000 paediatrics doses (50 mg artesunate) and 1,133,000 adult doses (100 mg artesunate) procured as part of round-two GFATM. The drug (under the brand name 'ARTECOSPE') has been distributed into 466 health facilities in 11 states out of Sudan 25 states free of charge. Unicef agreed to procure 1400,000 doses to be use in states where the GFATM is not there.

## Authors' contributions

EM Malik suggested the idea, drafted the paper and finalized the manuscript for submission. EM Malik, TA Mohamed, KA Elmardi, RM Mowien, AH Elhassan, SB Elamin and AA Mannan shared in collection of data, summarizing the findings and revision of the paper contents. ES Ahmed critically reviewed the paper.
